# Preliminary Assessment of Tara Gum as a Wall Material: Physicochemical, Structural, Thermal, and Rheological Analyses of Different Drying Methods

**DOI:** 10.3390/polym16060838

**Published:** 2024-03-19

**Authors:** Elibet Moscoso-Moscoso, Carlos A. Ligarda-Samanez, David Choque-Quispe, Mary L. Huamán-Carrión, José C. Arévalo-Quijano, Germán De la Cruz, Rober Luciano-Alipio, Wilber Cesar Calsina Ponce, Reynaldo Sucari-León, Uriel R. Quispe-Quezada, Dante Fermín Calderón Huamaní

**Affiliations:** 1 Nutraceuticals and Biomaterials Research Group, Universidad Nacional José María Arguedas, Andahuaylas 03701, Peru; dchoque@unajma.edu.pe (D.C.-Q.); huamancarrionmary@gmail.com (M.L.H.-C.); jcarevalo@unajma.edu.pe (J.C.A.-Q.); german.delacruz@unsch.edu.pe (G.D.l.C.); rluciano@unaat.edu.pe (R.L.-A.); wcalsina@unap.edu.pe (W.C.C.P.); rsucari@unah.edu.pe (R.S.-L.); uquispe@unah.edu.pe (U.R.Q.-Q.); dante.calderon@unica.edu.pe (D.F.C.H.); 2 Research Group in the Development of Advanced Materials for Water and Food Treatment, Universidad Nacional José María Arguedas, Andahuaylas 03701, Peru; 3 Department of Education and Humanities, Universidad Nacional José María Arguedas, Andahuaylas 03701, Peru; 4 Agricultural Science Faculty, Universidad Nacional de San Cristobal de Huamanga, Ayacucho 05000, Peru; 5 Administrative Sciences Faculty, Universidad Nacional Autónoma Altoandina de Tarma, Junín 12731, Peru; 6 Social Sciences Faculty, Universidad Nacional del Altiplano, Puno 21001, Peru; 7 Engineering and Management Faculty, Universidad Nacional Autónoma de Huanta, Ayacucho 05000, Peru; 8 Agricultural and Forestry Business Engineering, Universidad Nacional Autónoma de Huanta, Ayacucho 05000, Peru; 9 Ambiental Engineering, Universidad Nacional San Luis Gonzaga, Ica 11001, Peru

**Keywords:** tara gum, wall material, encapsulation, physicochemical properties, thermal properties, structural properties, rheological properties

## Abstract

Tara gum, a natural biopolymer extracted from *Caesalpinia spinosa* seeds, was investigated in this study. Wall materials were produced using spray drying, forced convection, and vacuum oven drying. In addition, a commercial sample obtained through mechanical methods and direct milling was used as a reference. The gums exhibited low moisture content (8.63% to 12.55%), water activity (0.37 to 0.41), bulk density (0.43 to 0.76 g/mL), and hygroscopicity (10.51% to 11.42%). This allows adequate physical and microbiological stability during storage. Polydisperse particles were obtained, ranging in size from 3.46 µm to 139.60 µm. Fourier transform infrared spectroscopy characterisation confirmed the polysaccharide nature of tara gum, primarily composed of galactomannans. Among the drying methods, spray drying produced the gum with the best physicochemical characteristics, including higher lightness, moderate stability, smaller particle size, and high glass transition temperature (141.69 °C). Regarding rheological properties, it demonstrated a non-Newtonian pseudoplastic behaviour that the power law could accurately describe. The apparent viscosity of the aqueous dispersions of the gum decreased with increasing temperature. In summary, the results establish the potential of tara gum as a wall material applicable in the food and pharmaceutical industries.

## 1. Introduction

The quest for natural polymers has intensified interest in unconventional plant resources [1,2,3]. Among these, tara gum extracted from the seeds of *Caesalpinia spinosa* stands out as a promising polymer. Tara gum has become a valuable source of polysaccharides, boasting advantageous physicochemical and functional properties, making it a material of growing interest in polymer science and food applications [4,5,6]. Its potential as an encapsulating agent has been acknowledged in diverse industry applications [5,7,8], attributed to its capacity for enhancing active compounds’ stability and controlled release. The increasing significance is underscored by the abundant gum production in Peru, accounting for 80% of the world’s total [6], driving the need for an exhaustive characterisation to take full advantage of this resource.

Only a few natural biopolymers act as encapsulation matrices [9,10]; the most common are gum arabic, maltodextrin, xanthan gum, and emulsifying starches, which are more widely used as encapsulating agents for food applications [11,12]. Therefore, it is necessary to evaluate the characteristics of tara gum since little is known about its physicochemical, structural, thermal, and rheological properties. It also represents a promising and relatively unexplored area of study on polymers and encapsulating materials in the current research context [7]. It is widely available in Peru and produces pods of high economic value due to the tannins in the leaflets and the gum in the endosperm of the seeds [13]. Conversely, various starches, proteins, maltodextrins, and gums are employed as wall materials for encapsulating diverse bioactive compounds using various techniques [1,14,15,16,17]. Understanding this gum’s structure and physicochemical properties can contextualise it within current encapsulant options, identifying its advantages and possible areas for improvement. This approach contributes significantly to advancing the field by expanding the repertoire of available materials and promoting sustainable solutions [18,19].

While tara gum holds encapsulating potential, its behaviour under physicochemical, structural, thermal, and rheological analyses remains to be thoroughly explored. This study comprehensively characterises fundamental properties: yield, moisture, water activity, hygroscopicity, bulk density, colour, ζ-potential, particle size, polydispersity, structure, elemental composition, functional groups, thermal stability, and flow behaviour. The results provide a rigorous understanding of the essential properties of tara gum and confirm its potential as an encapsulating material for multiple industrial applications. The main objective is to lay a solid scientific foundation to use this local resource in encapsulation, promoting regional circular economy and sustainability.

## 2. Materials and Methods

### 2.1. Materials

The tara pods used were kindly provided by farmers in the district of Talavera, province of Andahuaylas, Apurimac Region in Perú (coordinates 13°40′17.11″ S, 73°25′0.39″ W). Likewise, a commercial organic sample of tara gum, produced by the company Associated Mills SAC, was used for comparative purposes. We also used absolute ethanol (Scharlau, Sentmenat, Spain), sodium chloride (Spectrum Chemical Mfg. Corp, Bathurst, NB, Canada), and potassium bromide IR-grade (Thermo Fisher Scientific, Garfield, NJ, USA).

### 2.2. Obtaining the Tara Gum

A total of 120 g of germ-free tara seeds were placed in 800 mL of distilled water and stirred using an M6 thermomagnetic stirrer (CAT, Ballrechten-Dottingen, Germany) at 70 °C for 12 h to obtain a viscous substance. The extract was then filtered, purified, and mixed with 96% ethanol in a 1:1 ratio. This process was carried out to precipitate the viscous substance [20,21].

Subsequently, the purified extract was separated into three parts and subjected to a drying process using three methods. In the first method, a mini spray dryer B-290 (BÜCHI Labortechnik AG, Flawil, Switzerland) was used, with an inlet temperature of 100 °C and a pumping rate of 20%. The second method used forced convection oven drying FED 115 (BINDER, Tuttlingen, Germany) at 60 °C for 24 h. In the third method, a vacuum oven VD56 (Binder, Tuttlingen, Germany) was used at 30 °C and a pressure of 10 mBar.

**Figure 1:**
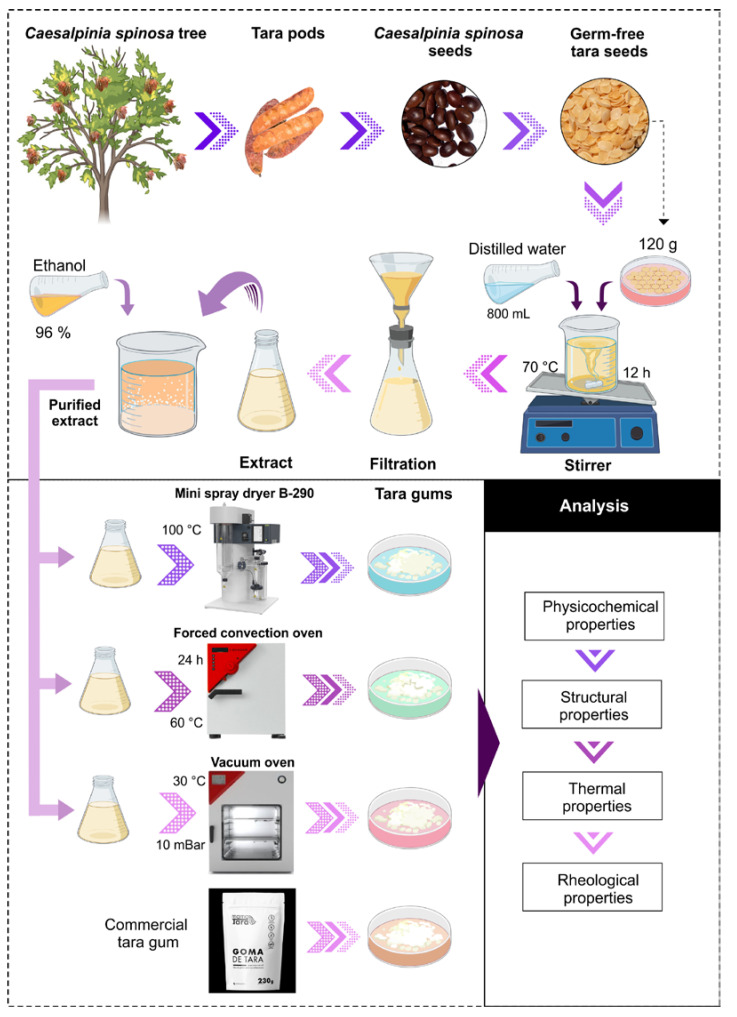
Experimental flow diagram.

Finally, the material obtained was collected in low-density polyethylene bags and stored in a desiccator at 20 °C for later use. Likewise, a commercial sample of tara gum produced by the company Associated Mills SAC was acquired and used for comparative purposes, which was obtained through the mechanical processes of the separation and milling of the endosperm from the tara seed, being considered a natural and gluten-free product, used as a thickener in the food industry. An experimental flow diagram is shown in Figure 1.

### 2.3. Yield

The yield was calculated based on the initial mass of the tara seeds used, and the final mass obtained after the extraction and purification was used to determine the efficiency of obtaining tara gum. The yield was expressed as a percentage and determined using the ratio between the initial and final mass, according to the following equation [22]:

(1) $$Y\;\left(\%\right)=\frac{mi}{mf}\times 100\;$$

where $Y\;\left(\%\right)$ is the yield in percent, mi is the initial mass (g), and mf is the final mass (g).

### 2.4. Moisture

Moisture was determined according to the methodology of AOAC 950.10. A total of 2 g of sample was weighed into watch capsules and placed inside a forced convection oven FED 115 (BINDER, Tuttlingen, Germany) at a controlled temperature of 105 °C until a constant weight was reached. Moisture content was expressed as the percentage ratio of the sample weight after drying to the sample weight before drying [23].

### 2.5. Water Activity (Aw)

To determine the Aw of the tara gum samples, specialised equipment known as a water activity meter was used. In particular, the HygroPalm23-AW model from Rotronic (Bassersdorf, Switzerland) was used. Before the measurements, the equipment was calibrated according to the manufacturer’s instructions. This ensured the reliability of the Aw readings as the meter was adjusted with standard solutions. Tara gum samples were placed in the reading chamber of the equipment, which measures the equilibrium relative humidity on the sample. This relative humidity value is converted to Aw according to the product characteristic curves stored in the meter’s memory [24].

### 2.6. Hygroscopicity

A total of 1 g of the sample was dispersed in 100 mL of a saturated NaCl solution (with a relative humidity of 75%) in an airtight container. The temperature was kept constant at 25 °C. After seven days, the samples were weighed again, and the results were expressed as a percentage increase in mass [25]:

(2) $$\%H=\left(\frac{m3-m2}{m2-m1}\right)\times 100$$

where %H is the mass increase in percent, m1 is the weight of the empty Petri dish, m2 is the weight of the Petri dish + sample, and m3 is the weight of the Petri dish + sample after seven days.

### 2.7. Bulk Density

The displaced volume method was used to determine the bulk density of the tara gum samples. This consists of weighing a known amount of the powdered sample and placing it in a 10 mL graduated cylinder. The graduated cylinder was gently tapped on a smooth, firm surface to settle the powder. The tapping removed the voids between the particles. Finally, the volume displaced by the sample after tapping the graduated cylinder was recorded. With the known mass and the measured volume, the bulk density (g/mL) was calculated using the mass/volume ratio [26].

### 2.8. Colour Analysis

A CR-5 colorimeter from Konica Minolta (Tokyo, Japan) was used, which allows the rapid and objective measurement of different colorimetric parameters based on the interaction of the sample with a standard light source. Before the measurements, the colorimeter was calibrated with standard black and white reference plates to ensure reliable readings. Then, powdered tara gum samples were successfully placed inside the reading cell, and the lightness values (L*), chroma a*, and chroma b* were recorded [27,28].

### 2.9. ζ-Potential

The dynamic laser light scattering technique was used to evaluate colloidal stability using ζ-potential measurements. Powdered samples (20 mg) were dispersed in ultrapure water (50 mL) and subjected to ultrasonication for 60 s before readings to ensure adequate particle distribution. Measurements were performed with a Zetasizer ZSU3100 (Malvern Instruments, Worcestershire, UK) at 25 °C using a specific DTS1080 cell to read the ζ-potential of the dispersions. This electrokinetic analysis allowed the prediction of the stability of the polymer suspensions due to the magnitude of the repulsive forces between the particles [29].

### 2.10. Particle Size and Polydispersity

To determine the particle size and polydispersity using a laser light scattering method, the sample particles were dispersed in isopropanol and subjected to ultrasound for 30 s. Then, a 600 nm helium–neon laser was incident on the sample, and the resulting diffraction pattern was measured using a Mastersizer 3000 instrument (Malvern Instruments, Worcestershire, UK) [30]. The polydispersity was determined using the amplitude index [31,32] according to the following relationship:

(3) $$Span\;index=\frac{D\;\left(90\right)-D\;\left(10\right)}{D\;\left(50\right)}$$

where $D\;\left(10\right)$, $D\;\left(50\right)$, and $D\;\left(90\right)$ correspond to the diameters relative to 10%, 50%, and 90% of the cumulative size distribution.

### 2.11. SEM-EDS Analysis

Morphological and elemental analyses were carried out using a scanning electron microscope (SEM) Prism E model (Thermo Fisher, Waltham, MA, USA). To prepare the tara gum samples, they were arranged on carbon adhesive tape, ensuring uniform distribution. Photomicrographs were captured at 30 kV, 2500×, and 500× magnification under low-vacuum conditions, allowing a detailed evaluation of the surface morphology. In addition, the elemental analysis was carried out using X-ray energy dispersion (EDS) [33].

### 2.12. FTIR Analysis

The functional groups of the tara gums were analysed using a Fourier transform spectrophotometer (FTIR) model Nicolet IS50 (ThermoFisher, Waltham, MA, USA). Tablets were prepared with 1 mg of sample and 99 mg of KBr. The mixture was crushed and passed through a press at 10 tons [34]. For the readings, the transmission module was used in the range of 400 cm^−1^ to 4000 cm^−1^, making use of a resolution of 8 cm^−1^, 32 scans were carried out to ensure an accurate capture of the spectral information [26].

### 2.13. Thermal Analysis

A total of 10 mg of the sample was utilised for thermal stability analysis via thermogravimetry (TGA). The measurement was performed using a thermal analyser (TA Instrument, New Castle, DE, USA) with a heating rate of 10 °C/min. A differential scanning calorimeter (DSC2500, TA Instrument, New Castle, DE, USA) was also used with 2 mg of the sample. The temperature range was set from 0 to 250 °C, with a heating rate of 10 °C/min in a nitrogen atmosphere [22].

### 2.14. Rheological Analysis

The experimental samples were continuously tested through an MCR 702e MultiDrive rotational rheometer (Anton Paar GmbH, Austria). Flow curve measurements were determined using the CC27 concentric cylinder with a diameter of 26.661 mm and a length of 39.996, with a cup diameter of 29.914 mm at a shear rate of 1 to 300 s^−1^. They were then analysed using shear stress models for fluids through the power law, Bingham Plastic, and Herschel–Bulkley models [35]. Table 1 shows the details of the rheological models.

### 2.15. Statistical Analysis

Tukey’s multiple comparison test was used to evaluate possible significant differences between groups, with a confidence level of 95%, after analysing variance (ANOVA). This analysis allowed the variations between groups to be examined and statistical disparities to be accurately determined. All statistical analyses and graphical representations were realised using OriginPro 2024 software from Origin Lab Corporation, based in Northampton, MA, USA. This methodological approach provided a rigorous and comprehensive evaluation of the differences between groups, ensuring a robust interpretation of the results obtained.

## 3. Results and Discussions

### 3.1. Physical and Chemical Properties of Tara Gum

Table 2 presents the physicochemical properties of spray-dried tara gum (GA), forced convection oven-dried tara gum (GE), vacuum oven-dried tara gum (GV), and commercial tara gum (GC) for comparison. In terms of yield, the vacuum oven drying method (GV) yielded the highest value at 45.21%, indicating optimistic process efficiency. Moisture content ranged from 8.63% to 12.55% across different samples, with these values being considered adequate as low moisture promotes material stability [36]. Water activity (Aw) values ranged from 0.39 to 0.41, a desirable characteristic for encapsulating agents due to increased resistance to microbial growth and biochemical deterioration reactions [37]. Other authors reported water activity values of 0.55 for almond gum and 0.53 for gum arabic [38]. Hygroscopicity values below 13.41% were found, a fundamental value, considering that the wall materials must present inert properties toward the active ingredients of the core, which must also be non-hygroscopic [39]. Hygroscopicity is a critical factor affecting the stability of food products [32]. Powders with values above 15.1% are considered highly hygroscopic [40]. On the other hand, powders with hygroscopicity values below 10% are considered non-hygroscopic and between 10.1% and 15% slightly hygroscopic, determined at a relative humidity of 75% [41]. Also, hygroscopicity values were reported for gum arabic between 26% and 40%, where fine particles (37 μm) with the highest value of 40% presented a high capacity to retain and adsorb water [42]. Concerning bulk density, values between 0.43 and 0.76 g/mL were found, which coincide with the densities of xanthan gum (0.46 g/mL) [43] and gum arabic (0.50 g/mL) [37].

The lightness values (L*) indicated that the spray-dried tara gum sample (GA) was the lightest, indicating a better preservation of the original characteristics through this technique, while the oven-dried samples (GE and GV) showed some darkening; similar values were obtained for gum arabic [37]. Regarding the tendency toward reddish (positive a* values) or greenish (negative a* values) tones, the GA sample presented a slightly reddish tone, unlike the rest; the commercial sample (GC) was the one with the highest greenish intensity. In the case of gum arabic, the value recorded for this parameter was 2.48 [37]. Yellow tones (positive b* values) were observed in all samples; other authors have reported values of 2.48 to 3.39 and 11.62 to 14.29 for gum arabic and almond gum, respectively [37].

The zeta potential values were negative in all tara gum samples due to ionisable acidic groups in their structures [22,44]. The zeta potential represents the potential difference between the compact ion layer and the dispersant medium, determining the repulsive force between the particles and their colloidal stability [45]. All the gums presented moderate stability between −16.57 and −23.77 millivolts [46].

The data indicate that tara gum is favourable for developing spray-drying encapsulation processes, avoiding the agglomeration of the encapsulating particles [22,30,47]. In food applications, adequate colloidal stability improves the interaction with the charged components of the matrix. The results are similar to those of commercial samples of tara gum used in spray-drying encapsulation processes [30,47]. The negative values suggest its usefulness in food and pharmaceutical products, promoting favourable interactions with its negative charge [5]. In addition to zeta potential, pH, ionic strength, and additives influence colloidal stability, requiring a more comprehensive approach for optimising the functional properties of these gums in various food and pharmaceutical applications [48].

The GA sample obtained by spray drying had the most petite particle sizes with a D10 of 1.71 µm, D50 of 3.46 µm, and D90 of 7.84 µm. This indicates that 90% of the particles were below 7.84 µm, with a mean of 3.46 µm and a minimum of 1.71 µm. This narrow distribution of sizes less than 10 microns makes the GA sample the most suitable for forming the walls of microcapsules, whose sizes vary between 1 and 100 µm. In contrast, the GV sample obtained by vacuum drying exhibited the largest sizes with a D90 of 375.40 µm and an average of 115.80 µm. Considering that the size of the nanostructures is located between the range of 1–1000 nm, this sample would not be helpful as a wall material for the nanoencapsulation process, for which the GA and GE samples with a D50 below 50 µm seem to be more viable. Regarding polydispersity, the GA sample showed the lowest index of 1.77, while GE and GV were the most polydisperse with indexes of 2.88 and 3.09, respectively. This reflects that spray drying and convection oven drying methods give rise to more homogeneous distributions of particle sizes [1,49].

Polydispersity constitutes an important indicator that describes the homogeneity of particle size distributions [45] for tara gum samples. This shows that most of the samples studied presented heterogeneous size distributions with significant variations between the most extensive and most minor fractions, according to the parameters D90 and D10, respectively. However, it should be noted that the tara gum sample obtained by spray drying (GA) exhibited the lowest polydispersity index of 1.77. This indicates that using this technique makes it possible to obtain the narrowest and most homogeneous distribution of particle sizes compared to the other methods evaluated. Higher polydispersity values were obtained for xanthan gum with 9.70 [50]. The results show that the tara gum obtained by spray drying has the most suitable characteristics to be used as a wall material in micro- and nanoencapsulation processes.

The results shown above show that tara gum’s physicochemical properties vary according to the drying method used. Spray-dried tara gum presented the best characteristics for use as an encapsulant. Spray drying allows for the quick drying of droplets and the formation of tiny, uniform spherical particles [51,52]. This method prevents browning and thermal degradation with convection and vacuum drying [53,54]. Therefore, choosing the drying method is a crucial factor that must be carefully considered during the production of gums for encapsulation applications [55]. However, further research is necessary to optimise the drying parameters to obtain gums with ideal physicochemical properties. In conclusion, this study provides valuable information to guide the selection and production of tara gums as wall materials in encapsulation processes.

### 3.2. SEM-EDS Analysis

**Figure 2:**
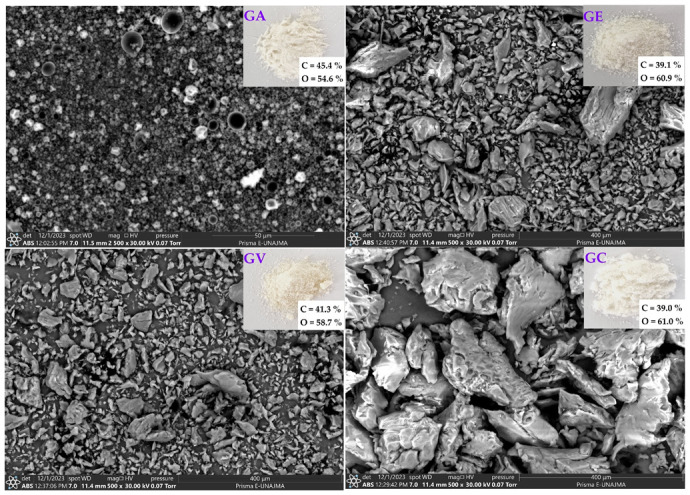
Scanning electron microscopy analysis on spray-dried tara gum (GA), forced convection-dried tara gum (GE), vacuum-dried tara gum (GV), and commercial tara gum (GC).

Figure 2 shows microphotographs of the tara gum samples, which presented variability in shape and size. Sample GA was observed at a magnification of 2500× (scale bar of 50 µm), and GE, GV, and GC were observed at 500× (scale bar of 400 µm). The GA sample showed a homogeneous surface without large visible pores and with mostly spherical shapes. Concerning GE, a primarily smooth topography with defined particles was observed. At the same time, GV exhibited an irregular surface, with numerous pores distributed over the particles, and some angular edges were distinguished, denoting fragility. GC showed a heterogeneous appearance with some aggregated particles, in which a wide distribution of particle sizes can be distinguished.

GA presented particles with a smaller size and a more homogeneous shape; the sizes of the particles can influence the dissolution rate of powdered gums [56], which in turn directly influences the intrinsic viscosity and molecular mass [57]. Generally, the dissolution rate of polysaccharide powders increases with a reduction in particle size [37,56]. Likewise, the exudates’ gums have various shapes and sizes [37,57].

Likewise, the C and O surface contents were analysed via energy-dispersive X-ray spectroscopy (EDS). It was observed that the carbon content varied between 39% and 45.4%, and the oxygen content varied between 54.6% and 61%. No significant differences in the C and O compositions were observed between the gums. EDS analysis confirmed that the different tara gum samples possess a similar composition of carbon and oxygen, which are chemical elements in biopolymers used as wall materials [26,58,59]. This verifies that the obtained gums are suitable with wall materials in micro- and nanoencapsulation processes. The different drying methods used do not alter this desired composition.

SEM and EDS analyses show that the drying method affects the morphology of tara gum. Spray drying achieves homogeneous spherical particles ideal for encapsulation, as water’s rapid evaporation fixes the droplets’ spherical shape [60]. Convective and vacuum methods cause irregularities due to higher thermal exposure [61]. The C and O composition remains stable, indicating that the chemical nature does not change. The drying conditions must be optimised to obtain the desired microstructure while preserving the composition.

### 3.3. FTIR Analysis

**Figure 3:**
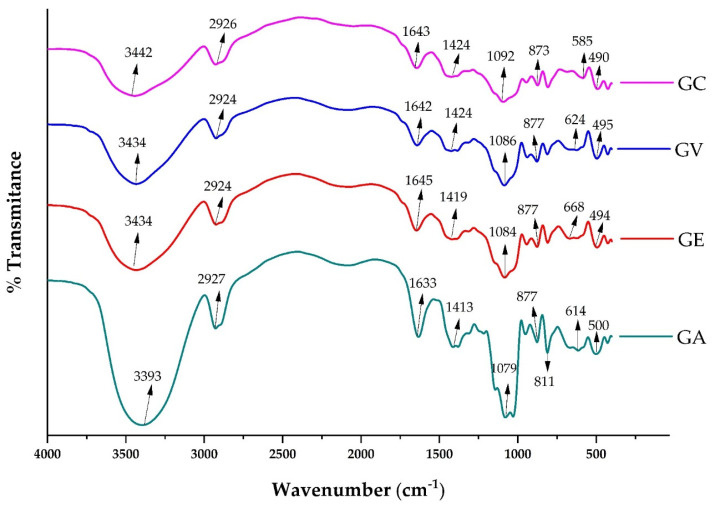
FTIR spectra of commercial tara gum (GC), vacuum-dried tara gum (GV), forced convection-dried tara gum (GE), and spray-dried tara gum (GA).

The infrared spectra of the gums obtained through spray drying, a forced convection oven, a vacuum oven, and commercially are shown in Figure 3. The four samples presented similar absorption patterns, indicating a similar chemical composition based on polysaccharides. Characteristic bands of polysaccharides such as hydroxyl (–OH), carbonyl (C=O), and C–O and O–H bonds were observed. Validated methodology was used to analyse and interpret the infrared (IR) spectra [62].

The IR spectra of the tara gum samples showed a broad absorption band at 3393–3442 cm^−1^ due to the stretching frequency of the OH group [63,64]. The presence of –OH stretching could be attributable to sugars such as galactose and arabinose [5]. The absorption band between 2924 and 2926 cm^−1^ is attributable to the stretching of the CH of the alkyl group [37,65], and those located between 1413 and 1424 cm^−1^ are due to the bending of the CH of the methyl group [64,65]. Likewise, the absorption bands located between 1633 and 1643 cm^−1^ are attributable to the asymmetric stretching of the carboxylate ion [65]. The bands located between 1079 and 1092 cm^−1^ corresponded to the presence of galactans [37]. Finally, the bands lying between 494 and 668 cm^−1^ were attributed to skeletal mode vibrations of the pyranose rings [37]. The FTIR analysis showed that tara gum’s chemical composition is preserved regardless of the drying method. The characteristic bands of functional groups are maintained, indicating that the polysaccharide structure is not significantly altered.

### 3.4. Thermal Analysis

**Figure 4:**
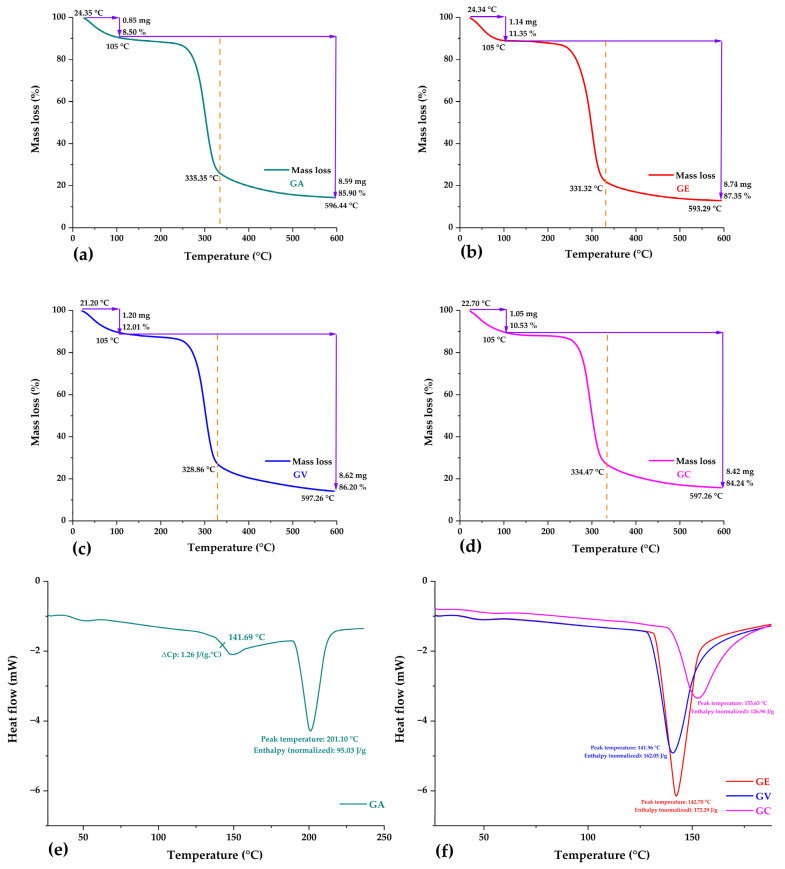
Thermogravimetric analysis: (**a**) spray-dried tara gum GA, (**b**) oven-dried tara gum GE, (**c**) vacuum-dried tara gum GV, (**d**) commercial tara gum GC, (**e**) DSC curves in GA, (**f**) DSC curves in GE, GV, and GC.

Graphical representations of the TG curves of all the samples of the tare gums are shown in Figure 4a–d. It can be noted that the samples exhibited similar thermal behaviours, evidencing the presence of mainly two events.

The first event occurred between 21.20 °C and 105 °C, with a mass loss between 8.50% and 12.01%, similar to the moisture content determined in the samples, which would correspond to the evaporation of free water due to the hydrophilic nature of the functional groups of the polysaccharides present in the polymeric matrix, this decrease is attributed to the onset of hydrogen bond breaking and the evaporation of water molecules, in addition to the elimination of other thermolabile compounds of low molecular weight [66,67].

A second event occurred between 105 °C and around 600 °C (with peaks around 328.86 °C and 335.35 °C), which was associated with a mass loss of between 84.24% and 87.35%, which is related to the thermal decomposition of the carbohydrates present in the tara gum [67,68]. At high temperatures, other organic compounds were eliminated in the gums, culminating in obtaining final residues [59,69,70]. 

Figure 4e illustrates the DSC analysis performed on all samples; in the specific case of the spray-dried tara gum (GA), an elevated glass transition temperature of 141.69 °C was obtained as a slight change in the slope of the curve [71]. This finding offers valuable insights into phase transitions and structural modifications within this material [33,72]. Glass transition temperature (Tg) is a critical parameter that influences the functional properties and encapsulation behaviours of polymeric materials, thus impacting their potential applications. A higher Tg can facilitate elevated temperatures during encapsulation processes, which is crucial for developing specific applications [22,30,47]. Similar glass transition temperatures have been reported for various polymeric wall materials used in spray-drying processes, as was the case for maltodextrin (155.34 °C) [72]; also, Kurozawa and Deschamps reported a Tg of 160 °C for maltodextrin [73,74], arabic gum (139.81 °C), native potato starch (138.26 °C) [22], quinoa starch (139.21 °C) [75], and spray-dried tara gum (157.7 °C) [22]. Glass transition temperatures below these values in the microcapsules and nanocapsules would indicate that encapsulation was successfully achieved [33,72].

In the cases of the GE, GV, and GC gums (Figure 4f), endothermic melting peaks of 142.70 °C, 141.36 °C, and 153.63 °C were observed. In addition, an absence of change in heat capacity between the initial and final state was observed, which was a limitation for determining the glass transition temperature in the present study. Therefore, further analysis is required for a complete understanding of the thermal processes present in these gums, as it is known that the glass transition temperatures of natural gums are usually high [22,30,47,75].

### 3.5. Rheological Properties

**Figure 5:**
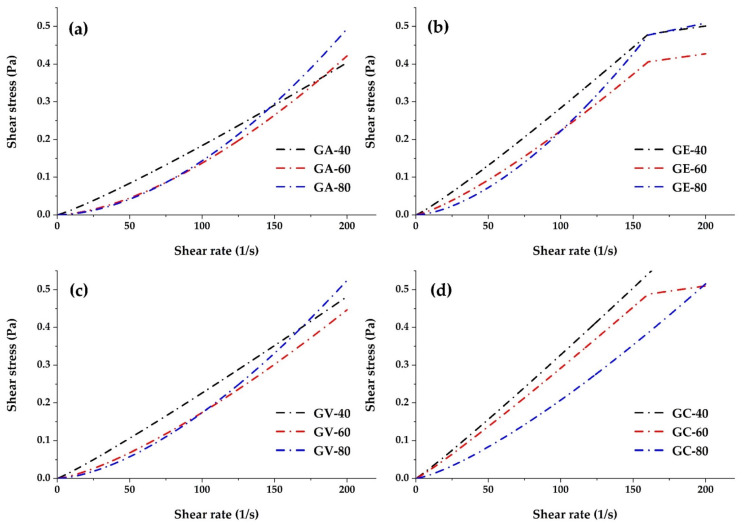
Shear rate and shear stress curves of spray-dried tara gum GA (**a**), oven-dried tara gum GE (**b**), oven vacuum-dried tara gum GV (**c**), and commercial tara gum GC (**d**) at 40, 60, and 80 °C.

Figure 5 shows the non-Newtonian rheological behaviours, with a nonlinear relationship between shear stress and strain rate. The GA sample recorded the highest maximum shear stress but one of the lowest maximum shear rates compared to the rest. The GE gum showed moderate values of these rheological parameters compared to the other samples. Sample GV showed similar strain rates to GA but significantly lowered maximum shear stress. Finally, GC exhibited the lowest levels of shear stress and maximum shear rate relative to GA, GE, and GV.

Within the common non-Newtonian nature, GC presented the most favourable rheological profile considering the magnitude of the experimentally determined parameters. Similar behaviours were reported for aqueous solutions of mucilage isolated from Opuntia ficus indica [76], which could be correlated with the shear rate using the power law model; likewise, locust bean gum showed non-Newtonian flow behaviour at speeds of high cut [77].

**Figure 6:**
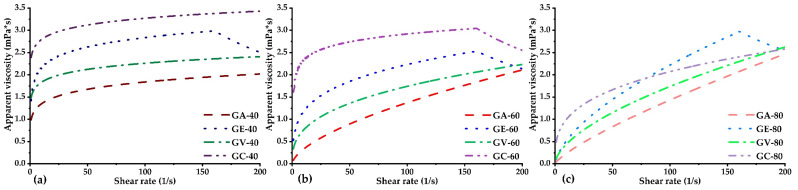
Apparent viscosity and shear stress curves at (**a**) 40 °C, (**b**) 60 °C, and (**c**) 80 °C.

In Figure 6, the apparent viscosity is not constant; as the temperature increases from 40 to 80 °C, the viscosities of all the samples decrease, with downwardly shifted curves being observed at higher shear rates. The observed behaviour is because increasing temperature reduces the intermolecular forces. This trend occurs because it contributes to increased intermolecular forces of attraction [5]. Similar behaviours were reported for Pithecellobium dulce, in which the viscosity of the gum decreased with increasing temperature, and the effect was more pronounced at temperatures below 50 °C [5]. The temperature study revealed that the tara gum samples under study show shear thinning behaviours at temperatures of 80 °C.

Apparent viscosity was observed to increase with the shear rate, this behaviour being typical in polymer solutions such as gums [78]. This phenomenon, observed in colloidal suspensions such as starch–water mixtures, is attributed to dissipative hydrodynamic interactions between particles, which induce the formation of hydroclusters [79].

This phenomenon, called dilatancy, causes changes in the structure and alignment of the polymer chains at high shear rates, increasing the interactions between chains and, therefore, the apparent viscosity of the fluid. This phenomenon explains the non-Newtonian behaviour and is a determining factor in the rheology of solutions of rubbers and other polymers [80]. The GE gum at all its temperatures and GC gum at 60 °C exhibit a particular rheological behaviour. An increase in apparent viscosity is observed at low shear rates, known as shear thickening or dilatation. However, as the shear rate exceeds 150 1/s, the apparent viscosity begins to decrease, a phenomenon called shear thinning. This behaviour is called rheopectic or rheopexy, where the viscosity versus shear rate curve forms a hysteresis loop [81]. A similar rheopectic behaviour has been reported for a maltodextrin-based thickener [82].

### 3.6. Rheological Analysis

The rheological models of the tara gum samples, obtained through atomisation, by forced convection drying, under vacuum, and as a commercial gum, are shown in Table 3. Non-Newtonian pseudoplastic rheological behaviour was observed (n < 1) for all gums at different temperatures. Most of the experimental data for tara gum fit the Herschel–Bulkley model very well, with R^2^ values greater than 0.9743. However, the initial shear stresses for GA, GE, GV, and GC were very close to zero; therefore, the power law model was the one that fit best with R^2^ values greater than 0.9739. This model describes the flow curves well, especially at high strain rates [83]. Xanthan gum, which is widely used as an encapsulating agent, fits the power law model [43,84], as did gum of diutan [84] and carboxymethylated tara gum [6], and has been used on numerous occasions to adjust the relationship between the apparent viscosity and the shear rate of biopolymer solutions [84].

### 3.7. Result Overview

A principal component analysis (PCA) of the physicochemical properties studied allowed for establishing relationships between these complex variables [85,86], providing a comprehensive view of their interactions. This methodology facilitated the identification of significant trends [47], which could help design and optimise obtaining tara gum with specific properties for use in the food, pharmaceutical, and cosmetic industries [30].

**Figure 7:**
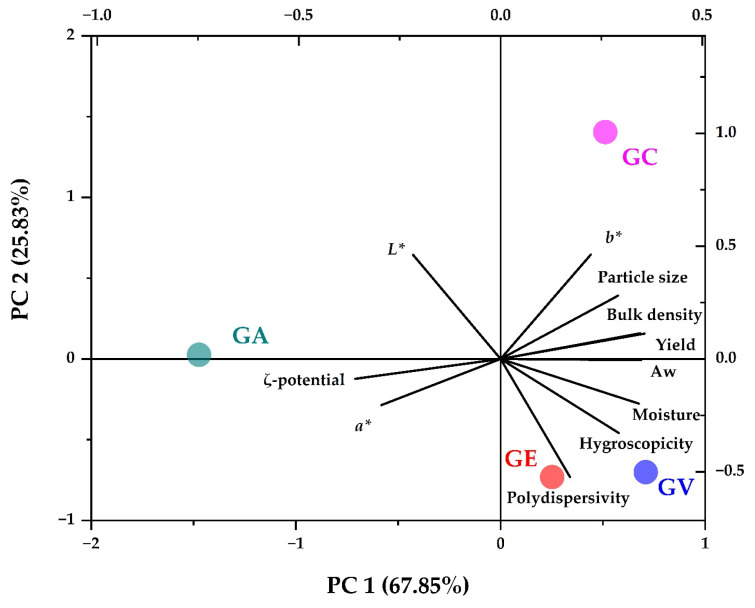
PCA study.

Figure 7 shows the PCA study, and it can be seen that spray-dried tara gum is associated with properties such as ζ-potential, luminosity L*, and chroma a*. These correlations suggest a possible interdependence between these properties, which could influence the stability and whiteness of tara gum. On the other hand, tara gums dried using a forced convection oven and vacuum oven (GE and GV) are more related to high values of moisture, Aw, hygroscopicity, and polydispersity, which would indicate that these gums are more prone to deterioration, and they present more significant heterogeneity in particle size. Finally, commercial tara gum (GC) is more related to high values of yield, particle size, apparent density, and chroma b*, which would indicate that in economic terms of the process, it would be more efficient, but on the contrary, due to the size of the particle, it would not be the most appropriate to use in the micro- and nanoencapsulation of compounds. Based on the results, spray-dried tara gum has the best physicochemical, structural, thermal, and rheological properties.

**Figure 8:**
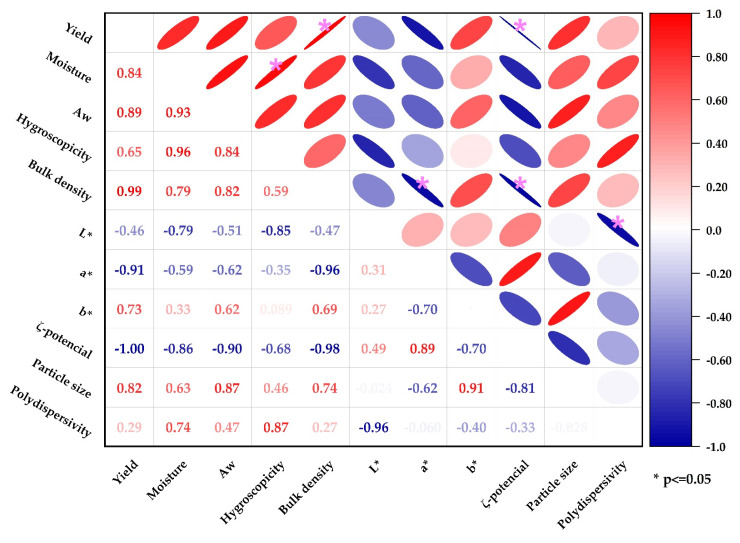
Pearson correlogram.

The Pearson correlogram (Figure 8) shows a significant positive correlation between hygroscopicity and moisture (r = 0.96), attributable to the more significant number of hydrophilic sites in the samples with higher residual water content [30]; a positive correlation was also observed between yield and bulk density (r = 0.99) since higher yields are obtained in denser and more compact particles [47]. On the other hand, significant negative correlations were found between yield and ζ-potential (r = −1.00), chroma a* and bulk density (r = −0.96), ζ-potential and bulk density (r = −0.98), and polydispersity and lightness (r = −0.96), which could be attributed to competitive interactions between the evaluated parameters. However, further studies are required to clarify the mechanisms involved. In conclusion, the correlation analysis revealed significant associations between the physicochemical properties of the studied gums.

## 4. Conclusions

Tara gum emerges as a promising natural polymer for applications as a wall material in encapsulation processes. The present work provides a comprehensive characterisation of tara gum in terms of physicochemical, structural, thermal, and rheological properties relevant to its application as a wall material. The tara gum obtained through different drying methods showed moisture contents between 8.63% and 12.55%, water activity between 0.37 and 0.41, hygroscopicity less than 13.41%, and apparent density between 0.43 and 0.76 g/mL. The FTIR analysis confirmed the presence of polysaccharides.

The spray-dried tara gum presented a better preservation of the original colour, smaller particle size, and better thermal properties. All samples showed non-Newtonian pseudoplastic rheological behaviour, adequately described by the power law. The apparent viscosity decreased with increasing temperature, showing shear thinning at 80 °C. The results position tara gum as a viable material for encapsulation, with favourable characteristics compared to other encapsulating agents. It is recommended that research be continued aimed at practical applications of encapsulation with tara gum as a wall material in the food and pharmaceutical industries.

## Figures and Tables

**Table 1:** Rheological models for non-Newtonian fluids.

Model	Equation	Parameters
Power Law	$\tau =k\gamma^{n}$	k, n
Bingham Plastic	$\tau =\tau_{y}+n_{B}\gamma$	$\tau_{y}$, $n_{B}$
Herschel–Bulkley	$\tau =\tau_{y}+k_{H}\gamma^{n}$	$\tau_{y}$, $k_{H}$, n

Note: τ is the yield strength (Pa), γ is the shear rate (s^−1^), k is the consistency index (Pa*s^n^), n is the behavioural index, $\tau_{y}$ is the yield strength (Pa), $n_{B}$ is the plastic viscosity (Pa*s), and $k_{H}$ is the consistency index (Pa*s^n^).

**Table 2:** Physical and chemical properties of tara gum obtained using different methods.

Properties	GA		GE		GV		GC	
	$\bar{x}\;\pm SD$	*	$\bar{x}\;\pm SD$	*	$\bar{x}\;\pm SD$	*	$\bar{x}\;\pm SD$	*
Yield (%)	12.18 ± 0. 45	a	42.38 ± 0.44	b	45.21 ± 0.60	c	51.37 ± 0.19	d
Moisture (%)	8.63 ± 0. 35	a	11.42 ± 0.40	bc	12.55 ± 0.48	c	10.86 ± 0.53	b
Aw	0.37 ± 0.01	a	0.39 ± 0.01	b	0.41 ± 0.01	b	0.40 ± 0.01	b
Hygroscopicity (%)	10.51 ± 0. 41	a	12.29 ± 0.43	b	13.41 ± 0.36	c	11.42 ± 0.16	d
Bulk Density (g/mL)	0.43 ± 0.01	a	0.71 ± 0.01	b	0.68 ± 0.01	c	0.76 ± 0.01	d
L*	91.02 ± 0.01	a	77.82 ± 0.01	b	79.62 ± 0.01	c	89.75 ± 0.01	d
a*	0.06 ± 0.01	a	−0.33 ± 0.05	b	−0.17 ± 0.04	c	−0.41 ± 0.01	b
b*	3.52 ± 0.05	a	4.23 ± 0.05	b	6.01 ± 0.05	c	9.83 ± 0.02	d
*ζ*-Potential (mV)	−16.57 ± 0.31	a	−22.29 ± 0.38	b	−22.97 ± 0.32	bc	−23.77 ± 0.29	c
Particle Size (µm)	3.46 ± 0.01	a	30.94 ± 0.79	b	115.80 ± 1.64	c	139.60 ± 0.89	d
Polydispersity	1.77 ± 0.01	a	2.88 ± 0.09	b	3.09 ± 0.05	c	1.54 ± 0.02	d

Note: $\bar{x}$ is the arithmetic mean, SD is the standard deviation, and different letters indicate significant differences per row evaluated for triplicates at 5% significance (*), the background color is the reference of the three chromatic coordinates. Spray-dried tara gum (GA), forced convection oven-dried tara gum (GE), vacuum oven-dried tara gum (GV), and commercial tara gum (GC).

**Table 3:** Rheological analysis.

Model	Parameters	GA			GE			GV			GC		
		40 °C	60 °C	80 °C	40 °C	60 °C	80 °C	40 °C	60 °C	80 °C	40 °C	60 °C	80 °C
Power Law	k (×10^−4^ Pa·s^n^)	9.9045	0.7980	0.3872	14.20	5.2710	0.9830	14.90	3.3455	1.1252	0.0024	16.10	4.8530
	n	1.1344	1.6180	1.7884	1.1073	1.2642	1.6141	1.0908	1.3583	1.5944	1.0684	1.0866	1.3152
	R^2^	0.9739	0.9854	0.9913	0.9912	0.9816	0.9884	0.9902	0.9796	0.9938	0.9965	0.9967	0.9926
Bingham Plastic	$\tau_{y}$(Pa)	0	0	0	0	0	0	0	0	0	0	0	0
	$n_{B}$(Pa·s^n^)	0.0020	0.002	0.0023	0.0025	0.0021	0.0024	0.0024	0.0022	0.0025	0.0034	0.0026	0.0025
	R^2^	0.9626	0.8578	0.8081	0.9826	0.9472	0.8628	0.9828	0.9244	0.8726	0.9944	0.9880	0.9422
Herschel–Bulkley	$\tau_{y}$(Pa)	0.0093	0.0166	0.0119	0.0034	0.0185	0.0215	0	0.0221	0.0219	0.0027	0.0035	0.0141
	$k_{H}\;$(×10^−4^ Pa·s^n^)	6.8532	0.2850	0.1993	0.0013	2.2715	0.3197	0.0017	1.1315	0.3804	0.0023	14.60	2.8672
	n	1.2010	1.8081	1.9070	1.1250	1.4179	1.8217	1.0672	1.5573	1.7947	1.0780	1.1040	1.4111
	R^2^	0.9743	1	1	0.9912	1	1	0.9892	1	0.9967	0.9966	0.9938	1

Note: τ is the yield strength (Pa), γ is the shear rate (s^−1^), k is the consistency index (Pa·s^n^), n is the behavioural index, $\tau_{y}$ is the yield strength (Pa), $n_{B}$ is the plastic viscosity (Pa*s), $k_{H}$ is the consistency index (Pa·s^n^), and R^2^ is the correlation coefficient. GA—spray-dried tara gum, GE—forced convection dried tara gum, GV—vacuum-dried tara gum, and GC—commercial tara gum (GC).

## Data Availability

They are available in the same article.
